# Anticoagulation with Argatroban in a Patient with Heparin-Induced Thrombocytopenia and Renal Insufficiency Undergoing Orthotopic Heart Transplantation

**DOI:** 10.1155/2021/9945225

**Published:** 2021-10-13

**Authors:** Michael Stuart Green, Johann Mathew, Christopher Ryan Hoffman, Henry Liu

**Affiliations:** ^1^Department of Anesthesiology, Thomas Jefferson University Hospital, Philadelphia, PA, USA; ^2^Department of Anesthesiology, Temple University Hospital, Philadelphia, PA, USA; ^3^Department of Anesthesiology, Penn State Milton S. Hershey Medical Center, Hershey, PA, USA

## Abstract

Unfractionated heparin is the anticoagulant of choice for cardiac surgery that requires cardiopulmonary bypass. However, it can cause serious side effects like heparin-induced thrombocytopenia (HIT), an immune-mediated process where antibodies are directed against heparin and platelet 4 complexes. In such cases, alternative pharmacologic strategies are implemented to facilitate safe bypass conditions. A woman with severe decompensated heart failure was heparinized for intra-aortic balloon pump and subsequent LVAD placement. On day 6, a fall in platelets from 113,000 to 26,000 was noted. She was diagnosed with HIT. Heparin was discontinued and replaced with an argatroban infusion for the duration of her care until heart transplantation was completed. We review the mechanism, diagnosis, and complications of HIT. We discuss cardiopulmonary bypass and its relation to heparin, HIT, and heparin alternatives. We discuss argatroban's relevant pharmacology, clinical use, advantages, and disadvantages.

## 1. Introduction

Patients undergoing cardiac surgery frequently require cardiopulmonary bypass (CPB) to isolate the heart for a motionless, bloodless surgical field. Anticoagulation is one of the key steps in preparing the patient for CPB. Unfractionated heparin (UFH) is the anticoagulant of choice for cardiac surgery and is used to prevent thrombin generation and hence prevent clotting in the extracorporeal circuit. However, it can cause serious side effects like heparin-induced thrombocytopenia. Type 2 HIT is an immune-mediated process where antibodies are directed against heparin and platelet 4 complexes (PF4), resulting in a >50% fall in platelets and thrombosis after 5–10 days of heparin therapy. Despite the resultant fall in platelets, patients present with an increased risk of thrombosis [1]. We present a case of new onset HIT with biventricular failure and acute renal failure for urgent heart transplantation, which elicited the problems one may face when using argatroban during CPB.

## 2. Case Summary

A 28-year-old woman with postpartum cardiomyopathy was admitted for severe decompensated heart failure (EF < 15%). Subsequently, she developed acute renal failure with creatinine levels rising from 1.0 to 2.9 mg/dL during hospitalization. She was started on inotropes and vasopressors, and intra-aortic balloon pump and LVAD were placed through heparinization. However, the patient continued to deteriorate requiring an urgent transplant. On day 6, a fall in platelets from 113,000 to 26,000 was noticed. Heparin was discontinued, and the patient was started on argatroban to attain aPTT 2-3 times baseline. After availability of a suitable donor, the patient was scheduled for heart transplant on day 10. Preoperatively, the labs were as follows: hemoglobin: 8 g/dl; platelets: 110000; creatinine: 2 mg/dl; PT: 10.9; and aPTT: 60.1. Given the timing and magnitude of the platelet count decline, along with its resolution after heparin cessation, the need for further laboratory investigation was deemed not necessary, and the patient was diagnosed with HIT. Two peripheral intravenous lines, 16G and 18G, and a 20G right radial arterial line were placed, followed by central venous catheterization. Intraoperatively, induction and intubation were uneventful. After sternotomy, a loading dose of argatroban 200 mcg/kg was administered to reach the target ACT > 400 (activated clotting time). ACT values were derived utilizing the point-of-care Hemochron Signature Elite, whole blood microcoagulation system. Once ACT >400, the patient was placed on CPB. This was followed with an infusion of argatroban at 3.5 mcg/kg/min initially and then titrated to 3 mcg/kg/min as guided by ACT. The infusion was continued until the patient was successfully weaned from CPB. During surgery, no clots were noted in the extracorporeal circuit, and postoperatively, there were no thrombotic and hemorrhagic complications. aPTT normalized 23 hours after stopping argatroban. The postoperative course was significant only for line sepsis requiring a prolonged course of antibiotic therapy and oliguria for which the patient was being hemodialyzed prior to surgery.

## 3. Discussion

HIT is a potentially life-threatening immune-mediated disorder that can lead to widespread thrombosis in 1–3% of patients exposed to unfractionated heparin after cardiac surgery [2]. In type 2 HIT, a greater than 50% reduction in platelets is observed 5–10 days after heparin therapy and is closely associated with the initiation of thrombosis, and the platelet count begins to rise only after discontinuing heparin [3, 4]. The magnitude of fall in platelet count is an important risk factor for the development of thrombosis. Bleeding is not commonly seen in these patients, and the observed thrombocytopenia is due to the removal of activated platelets and antibody-coated platelets by the reticulo-endothelial system [3]. When clinical suspicion for HIT is high, in addition to stopping heparin, nonheparin anticoagulants should be started to prevent further development of thrombosis. The diagnosis of HIT is a clinicopathological one, and various clinical scoring systems have been used to predict the likelihood of HIT [2]. Use of anti-PF4-heparin enzyme immunoassay and a functional test like serotonin-release assay increase the diagnostic accuracy for HIT [5]. Laboratory diagnosis, although confirmatory, may be delayed, and thus necessitates a clinical diagnosis of HIT.

These patients will require an alternative form of anticoagulation before going on bypass. Commonly used agents include argatroban, bivalirudin, fondaparinux, and danaparoid, and they act by inhibiting thrombin generation. However, their use can cause certain concerns in patients scheduled for cardiac surgery. The presence of acute kidney injury in this patient permitted only the use of argatroban as both lepirudin and danaparoid are mainly excreted in the kidneys [3]. The use of argatroban for CPB as an alternative to heparin has been explored in recent times, but there are many challenges in the form of lack of dosing guidelines and absence of an antidote. Currently, argatroban has been approved for use in HIT for percutaneous coronary intervention procedures, but its use for CPB is off label [6].

Argatroban is a small synthetic molecule, which inhibits both clot bound and free thrombin. It reaches steady state plasma quickly with a predictable dose-response effect and correlates with the desired anticoagulant effect [7]. It has a short half-life of about 45 minutes and allows early reversibility of the anticoagulation effect. However, hemodilution during CPB may alter the pharmacokinetics, making the response less predictable. This may lead to inadequate anticoagulation during CPB or excessive bleeding in the postoperative period. Other properties that make argatroban a suitable alternative are the lack of cross reactivity with HIT antibodies and that argatroban itself does not induce antibodies that could alter its clearance [7]. The efficacy of the drug is monitored with aPTT at low doses and ACT at higher doses. While ACT is commonly used to monitor anticoagulation during CPB, the ACT required to prevent clot formation is still unclear. There have been reports of clot formation in the circulation even with an ACT of 495 seconds, and it has been recommended that ACT be kept in the range of 500–600 seconds [8]. The commonly used tests such as aPTT and ACT have a curvilinear dose relationship with plasma direct thrombin inhibitor (DTI) concentration, making it difficult to predict the required level of anticoagulation [9]. This probably could be the reason for thrombosis during CPB even with ACT >400 seconds. In our case, we were able to maintain an ACT >400 seconds without any evidence of clotting in the extracorporeal circuit. Factors such as hemodilution and hypothermia besides argatroban concentration in the circulation influence the ACT values. Keeping the ACT at a higher range could be a probable solution to prevent clot formation, but it runs the risk of increased chances for postoperative bleeding because of the longer duration required for ACT to normalize [6]. It takes 2–4 hours for the aPTT to normalize after discontinuing the drug, but in our case, it took about 23 hours probably because of the altered hemodynamics during CPB. However, it had little bearing on bleeding in the postoperative period. DTI specific assays like Ecarin clotting time (ECT) have shown to better correlate with DTI plasma concentration than ACT [10] and have shown intraoperative ECT monitoring during CPB may be a safer alternative. Another disadvantage with using argatroban is the absence of an antidote in situations of life-threatening bleeding during surgery and in the postoperative period. In such situations, the drug should be discontinued and the patients should receive symptomatic treatment. To counter the deficiency caused by a lack of antidote, other alternatives like using unfractionated heparin (UFH) with prostacyclin, a potent platelet aggregation inhibitor, have been explored. However, the use of prostacyclin was associated with significant hypotension [11]. A newer option where UFH was combined with tirofiban, a short-acting GP IIb–IIIa inhibitor, has shown to be promising with no evidence of thrombosis or excessive hemorrhaging. In patients with a previous episode of HIT, the use of heparin is considered a safe option after the platelet activating antibodies disappear. In an urgent scenario, therapeutic plasma exchange has been used to reduce antibody titres prior to the procedure.

While the use of argatroban in our case had a successful outcome, there have been reports illustrating various complications in the perioperative period with the use of this medication [6]. More studies will be needed to determine ideal dosage guidelines, and appropriate monitoring for the use of argatroban as a heparin alternative in those patients undergoing CPB with a HIT is needed.
